# Molecular Evolution of the Fusion Protein (*F*) Gene in Human Respirovirus 3

**DOI:** 10.3389/fmicb.2019.03054

**Published:** 2020-01-15

**Authors:** Jumpei Aso, Hirokazu Kimura, Haruyuki Ishii, Takeshi Saraya, Daisuke Kurai, Yuki Matsushima, Koo Nagasawa, Akihide Ryo, Hajime Takizawa

**Affiliations:** ^1^Department of Respiratory Medicine, School of Medicine, Kyorin University, Tokyo, Japan; ^2^Department of Health Science, Graduate School of Health Science, Gunma Paz University, Gunma, Japan; ^3^Department of Microbiology, School of Medicine, Yokohama City University, Kanagawa, Japan; ^4^Department of General Medicine, Division of Infectious Diseases, School of Medicine, Kyorin University, Tokyo, Japan; ^5^Division of Virology, Kawasaki City Institute for Public Health, Kanagawa, Japan; ^6^Department of Pediatrics, Graduate School of Medicine, Chiba University, Chiba, Japan

**Keywords:** molecular evolution, human respirovirus 3, fusion protein (*F*) gene, fusion peptide, conformational epitope

## Abstract

To elucidate the evolution of human respirovirus 3 (HRV3), we performed detailed genetic analyses of the *F* gene (full-length) detected from hundreds of HRV3 strains obtained from various geographic regions. First, we performed time-scaled evolutionary analyses using the Bayesian Markov chain Monte Carlo method. Then, we performed analyses of phylodynamics, similarity, phylogenetic distance, selective pressure, and conformational B-cell epitope with the F-protein structural analyses. Time-scaled phylogenetic tree showed that the common ancestor of HRV3 and bovine respirovirus 3 diverged over 300 years ago and subdivided it into three major clusters and four subclusters during the most recent 100 years. The overall evolutionary rate was approximately 10^–3^ substitutions/site/year. Indigenous similarity was seen in the present strains, and the mean phylogenetic distance were 0.033. Many negative selection sites were seen in the ectodomain. The conformational epitopes did not correspond to the neutralizing antibody binding sites. These results suggest that the HRV3 *F* gene is relatively conserved and restricted in this diversity to preserve the protein function, although these strains form many branches on the phylogenetic tree. Furthermore, HRV3 reinfection may be responsible for discordances between the conformational epitopes and the neutralizing antibody binding sites of the F protein. These findings contribute to a better understanding of HRV3 virology.

## Introduction

Human respirovirus 3 (HRV3, formerly called human parainfluenza virus 3) is a negative-sense stranded RNA virus belonging to the genus *Respirovirus* in the family *Paramyxoviridae*. HRV3 causes acute respiratory diseases, such as common colds, croup (acute laryngotracheobronchitis), bronchiolitis, and pneumonia, and is distributed worldwide as the most prevalent type of former human parainfluenza viruses, such as HRV1 (formerly called human parainfluenza virus 1) or human orthorubulaviruses 2 and 4 (formerly called human parainfluenza viruses 2 and 4) (Henrickson, 2003; Weinberg et al., 2009; Karron and Collins, 2013; Abedi et al., 2016; Maykowski et al., 2018). The virus mainly infects infants, and approximately 80% of children experience infection by 4 years of age (Karron and Collins, 2013). However, some epidemiological studies have shown that HRV3 causes recurrent infections throughout life (Weinberg et al., 2009; Maykowski et al., 2018). Moreover, acute respiratory infection outbreaks due to HRV3 in immunocompromized patients have also been occasionally reported (Lee et al., 2011; Harvala et al., 2012). Therefore, HRV3, as well as human orthopneumovirus, formerly called HRSV, human respiratory syncytial virus, generates a disease burden of acute respiratory infection in all age groups in humans (Hall, 2001).

The HRV3 genome encodes six genes and translates them into eight proteins (Karron and Collins, 2013). Of these, two envelope proteins, i.e., the fusion protein (F protein) and hemagglutinin-neuraminidase (HN protein), play important roles not only as major antigens but also during host cell infection (Karron and Collins, 2013). In particular, the F protein presents as a homotrimer and mediates membrane fusion between the virus envelope and host cellular membrane (Moscona, 2005). Initially, the F protein protrudes from the virus envelope in a metastable prefusion state (F0 protein). This inactive precursor is cleaved at its activating proteolytic site by a host protease, generating the two subunits F1 and F2. A conformational change of the F1 + F2 proteins to the postfusion state results in membrane fusion (Chang and Dutch, 2012). The efficacy of these processes may contribute to the infectivity and pathogenicity of the virus (the detailed processes are shown in Supplementary Figure S1). Therefore, several mechanisms of HRV3 infection may be associated with the detailed structure and functions of the F protein as well as the molecular evolution of the *F* gene. To the best of our knowledge, however, the molecular evolution of this important protein remains unclear. Recent evolutionary analysis techniques using various bioinformatic technologies may enable us to elucidate these questions. Therefore, in this study, we performed detailed evolutionary analyses of the F protein full-length coding region of HRV3 strains collected from various geographic areas.

## Materials and Methods

### Strains Used in This Study

To understand more fully the molecular evolution of the HRV3 *F* gene, we comprehensively collected nucleotide sequences including the full-length coding region of the gene (position 4987–6603; 1,617 nt for HPIV3/BuenosAires/ARG/002/2017 strain, GenBank accession No. MG773276) from GenBank^1^ in March 2019. We selected strains with confirmed information of the detected/isolated years and regions. In addition, strains with ambiguous sequences (e.g., N, Y, R, and V) were omitted from the dataset, and 465 strains remained. Furthermore, among the three or more strains with similar sequences, two were chosen randomly and kept in the dataset, a process necessary for further phylogenetic analyses. Identical sequences were identified by Clustal Omega (Goujon et al., 2010; Sievers et al., 2011) and excluded. Finally, 377 strains remained, and we added a strain of bovine respirovirus 3 (BRV3, Shipping Fever strain, AF178655), which is the most closely related species to HRV3 among the families, as an outgroup from GenBank. All 378 strains used in the present study are shown in Supplementary Table S1. Multiple alignments for these nucleotide sequences were performed using MAFFT version 7 (Katoh and Standley, 2013), and the sequences were trimmed to 1,617 nt after the alignment. We uploaded the alignment file as Supplementary File S1.

### Time-Scaled Phylogenetic Analysis and Phylodynamic Analyses Using the Bayesian Markov Chain Monte Carlo Method

To examine the evolution of the HRV3 strains, we conducted a time-scaled phylogenetic analysis of full-length sequences of the HRV3 *F* gene using the Bayesian Markov chain Monte Carlo (MCMC) method in BEAST version 2.4.8 (Bouckaert et al., 2014). Before the molecular clock analyses, we verified whether our dataset consisted of sufficient genetic distance between sampling times to yield a statistical relationship between genetic divergence and sampling time. To evaluate such temporal signal of the sequences for reliable estimation, we utilized TempEst version 1.5.3 (Rambaut et al., 2016). Our dataset demonstrated a positive correlation between genetic divergence and sampling time and seemed to be appropriate for molecular clock analysis (data shown in Supplementary Figure S2). Next, for the selection of a suitable substitution model, the jModelTest 2.1.10 program (Darriba et al., 2012) was applied. The path sampling method (Lartillot and Philippe, 2006) was performed by the Path sampler implemented in BEAST to determine the best of four clock models (strict clock, exponential relaxed clock, relaxed clock log normal, and random local clock) and three tree prior models (coalescent constant population, coalescent exponential population, and coalescent Bayesian skyline). Using the obtained strains and the selected models, an MCMC tree was calculated by the BEAST software. To confirm convergence, Tracer version 1.7.1^2^ was used to evaluate effective sample sizes (ESS), and values above 200 were accepted. After burning in the first 10% of the trees, the maximum clade credibility tree was produced by TreeAnnotator version 2.4.8 in the BEAST package. The evolutionary rate of the collected *F* gene was calculated by the MCMC method simultaneously, and the values were confirmed in Tracer. The Bayesian MCMC phylogenetic tree was illustrated by FigTree version 1.4.0^3^, and the 95% highest posterior densities (HPDs) of all internal nodes were computed. Moreover, the clustering of the strains in the constructed phylogenetic tree of the HRV3 *F* gene followed the illustrated tree’s topology. We named the three clusters A–C, and five subclusters of cluster C were named C1–C5 as described previously (Tsutsui et al., 2017). We also measured the evolutionary rates of the 377 total HRV3 strains and strains of cluster C (including each dataset of subclusters C1, C3, and C5). TempEst version 1.5.3 was also applied for these datasets, and the results are presented in Supplementary Figure S2. The marginal likelihood values for the model selection and the parameters of the Bayesian MCMC analyses are outlined in Supplementary Tables S2, S3, respectively. Also, the statistics calculated by Tracer for each dataset are shown in Supplementary Tables S4–S9, and the BEAST XML files are provided as Supplementary Files S2–S7.

We also introduced the past genome population dynamics of the HRV3 *F* gene with Bayesian skyline plots (BSPs), generated with BEAST version 2.4.8, using a tree prior model of coalescent Bayesian skyline. The demographic histories of all 377 strains and the strains of subclusters C1, C3, and C5 were examined. Suitable substitution and clock models for each dataset were decided as defined above. The visualization of the demographic reconstructions was provided by Tracer version 1.7.1. The marginal likelihood values, parameters of the Bayesian MCMC analyses, and statistics computed by Tracer are shown in Supplementary Tables S2–S9.

### Similarity Plot Analysis and Calculation of Phylogenetic Distances

We used two different methods to estimate the phylogenetic diversity of the HRV3 *F* gene. Similarity plot analysis of the full-length *F* gene compared with the nucleotide sequence of a prototype strain (Washington/1957 strain) was performed using SimPlot version 3.5.1 (Lole et al., 1999). Similarity of the *F* genes was calculated using a window size of 50 nucleotides and a step size of 20 bp.

Furthermore, we analyzed phylogenetic distances among all HRV3 strains to study the range of evolution for the *F* gene. The phylogenetic tree of all HRV3 strains was constructed by the maximum likelihood (ML) method using MEGA7 software (Kumar et al., 2016), and the branch reliability was supported by 1,000 replications of bootstrap values. We applied the jModelTest 2.1.10 program to decide the best substitution model for the ML method. Subsequently, the phylogenetic distance of the ML tree was calculated using the Patristic program (Fourment and Gibbs, 2006).

### Selective Pressure Analyses

The selective pressure sites for the F protein of HRV3 were analyzed by calculating non-synonymous (*d*N) and synonymous (*d*S) substitution rates at each amino acid site using the Datamonkey^4^ web server. We used two algorithms to identify the selective pressure sites of the F protein. The Mixed Effects Model of Evolution (MEME) method (Murrell et al., 2012) was applied to predict sites of positive selection, which assumes that the pressure varies across branches. Alternatively, the Fast, Unconstrained Bayesian AppRoximation (FUBAR) method, which uses an approximate hierarchical Bayesian approach with an MCMC method (Murrell et al., 2013), was applied to infer negative selection sites. Evidence of selective pressure was supported by *p*-values of <0.05 for MEME and posterior probabilities of >0.9 for FUBAR.

### Analyses of Conformational B-Cell Epitopes and Amino Acid Substitution Sites With Mapping on the Three-Dimensional Structure of the HRV3 F Protein

To analyze accurately the pressure of human immune defense against the natural state of the HRV3 F protein, we predicted conformational epitopes on the trimeric prefusion state. Structural models of the prefusion F protein of HRV3 were constructed for representative strains from each group (prototype, Washington/1957 strain, S82195; subcluster C1, HPIV3/ARG/10068/2004 strain, KF530245; subcluster C3, LZ22 strain, FJ455842; subcluster C5, HPIV3/MEX/1099/2004 strain, KF687320) using MODELLER 9.22 (Webb and Sali, 2017). The templates for homology modeling were based on the crystal structure of the protein (Protein Data Bank accession ID: 6MJZ) found via the BLAST web server^5^. Amino acid sequences of each strain were aligned using MAFFTash version 4.1 (Standley et al., 2007; Katoh et al., 2009). Energy minimization of the generated structures was performed using GROMOS96 (Scott et al., 1999), implemented by Swiss PDB Viewer v4.1.0 (Guex and Peitsch, 1997). The models were assessed by Ramachandran plot analyses through RAMPAGE (Lovell et al., 2002). We analyzed conformational epitopes of the constructed models using DiscoTope 2.0 (Kringelum et al., 2012), BEpro (Sweredoski and Baldi, 2008), ElliPro (Ponomarenko et al., 2008), EPCES (Liang et al., 2007), and EPSVR (Liang et al., 2010) with cut-off values of -3.7 (DiscoTope 2.0), 1.3 (BEpro), 0.5 (ElliPro), and 70 (EPCES, EPSVR). Accuracy of the analyses was also supported among the consensus sites predicted by more than four of the five methods, and regions with close residues over two of the sites on the trimeric structure models were determined as conformational epitopes. We also estimated the amino acid substitution of the representative strains of each cluster and subcluster (which includes subclusters C1, C3, and C5) to the prototype strain. Finally, we mapped predicted B-cell epitopes and previously identified epitopes with amino acid substitution sites on the constructed prefusion F-protein models using UCSF Chimera Version 1.13 (Pettersen et al., 2004).

## Results

### Time-Scaled Evolutionary Analyses and the Phylodynamics of the HRV3 *F* Gene

**FIGURE 1:**
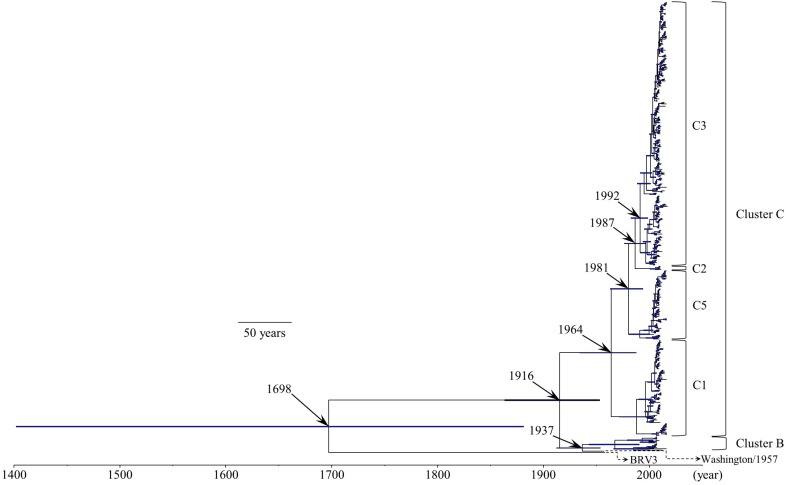
Time-scaled evolutionary tree of the full-length HRV3 *F* gene constructed by the Bayesian MCMC method. The scale bar represents time (year). Blue bars indicate the 95% highest posterior density (HPD) for each branch year.

We constructed a time-scaled evolutionary tree of the HRV3 *F* gene by the Bayesian MCMC method (Figure 1). The tree showed that the gene might have diverged from the common ancestor of BRV3 over 300 years ago (1698; 95% HPD, 1402–1882). Further division of three major clusters (A, B, and C) occurred around 1916 (95% HPD, 1864–1954). Moreover, the most recent common ancestor of cluster C might have emerged in 1964 (95% HPD, 1935–1988) and created four subclusters during these several decades (no strains belonged to subcluster C4 in this study). Of these, strains belonging to subcluster C3 are the most prevalent type at the present day. Also, the histograms indicating the distribution of the numbers of collected strains and sampling years for all strains (including each subcluster) are shown in Supplementary Figure S3.

Table 1 shows the evolutionary rates of the HRV3 *F* gene of all collected strains, with statistical analyses between rates of the clusters and subclusters. The mean rate (95% HPD) for the *F* gene of all HRV3 strains in the present study was estimated to be 9.40 × 10^–4^ (7.75 × 10^–4^–1.11 × 10^–3^) substitutions/site/year. Furthermore, the mean rate (95% HPD) of all strains belonging to cluster C was 8.80 × 10^–4^ (7.39 × 10^–4^–1.03 × 10^–3^) substitutions/site/year.

**TABLE 1:** Evolutionary rates of all 377 HRV3 strains and each cluster/subcluster.

	**Evolutionary rates (95% HPD) (substitutions/site/year)**
All HRV3 (377 strains)	9.40 × 10^–4^ (7.75 × 10^–4^–1.11 × 10^–3^)
Cluster C (364 strains)	8.80 × 10^–4^ (7.39 × 10^–4^–1.03 × 10^–3^)
Subcluster C1 (81 strains)	9.32 × 10^–4^ (6.75 × 10^–4^–1.19 × 10^–3^)
Subcluster C (221 strains)	8.81 × 10^–4^ (7.13 × 10^–4^–1.06 × 10^–3^)
Subcluster C5 (58 strains)	9.84 × 10^–4^ (6.77 × 10^–4^–1.32 × 10^–3^)

**FIGURE 2:**
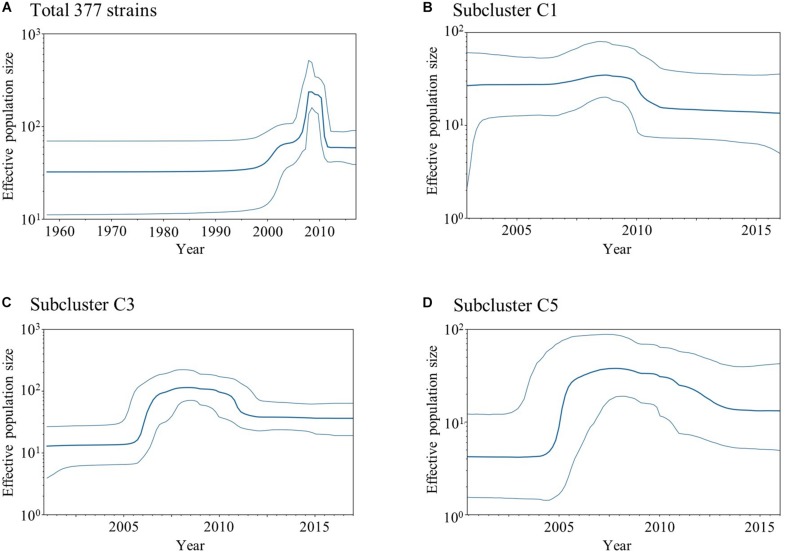
Plots of BSP analyses for the HRV3 *F* gene. Each panel illustrates the phylodynamics of all 377 strains **(A)**, subcluster C1 **(B)**, subcluster C3 **(C)**, and subcluster C5 **(D)**. *Y* and *x*-axes indicate the effective population size and time in years, respectively. The thick blue line shows the median value over time. 95% HPD intervals are represented by thin blue lines.

To estimate the past genome population size of the HRV3 *F* gene, we carried out BSP analyses of the collected HRV3 strains (Figure 2). As depicted in Figure 2A, the effective population size for the HRV3 *F* gene from all strains was inferred to be constant until 2000 and started to increase in size until 2010. However, the size decreased rapidly after 2010 and was constant thereafter. The size of the subcluster C1 strains gradually increased until 2010 and then declined (Figure 2B). The strains of subcluster C3 experienced a great increase in effective population size around 2006 (Figure 2C), slight decrease after 2010, and constant population ever since. The size of subcluster C5 strains fluctuated similarly to subcluster C3, which suddenly increased around 2005, gently declined after 2010, and remained constant after 2013 (Figure 2D). These results suggest that variations of the effective population size are specific for each variant, although the recent increase and decrease in the sizes of the strains for subclusters C1, C3, and C5 may reflect the phylodynamics of all HRV3 strains.

### Similarity Analyses

**FIGURE 3:**
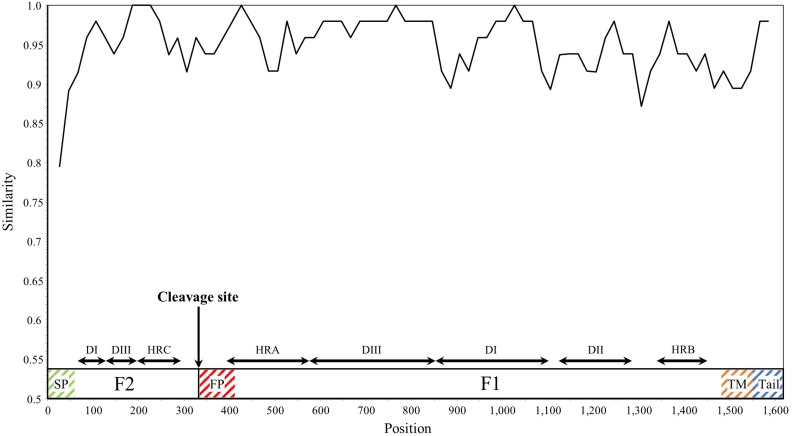
Plot of similarity analysis of the F gene across all HRV3 strains. Nucleotide similarity to the prototype strain (Washington/1957 strain) was evaluated using SimPlot. Nucleotide position numbers correspond to the F gene in the prototype strain. The cleavage site and the positions of each domains for the F1 and F2 subunits are shown below the graph (Yin et al., 2005). SP, signal peptide; DI–DIII, domains I–III; HRA–HRC, heptad repeat A–C; FP, fusion peptide; TM, transmembrane anchor; and Tail, cytoplasmic tail.

SimPlot analysis was performed for the HRV3 *F* gene in all collected strains (Figure 3). Overall, the similarity of the nucleotide sequences was over 90%, except for the signal peptide regions. Notably, the similarity of some junction sites (*cf.*, between the heptad repeat C and fusion peptide regions) was relatively low compared with the other regions. These results suggested that the *F* gene is conserved, but differences in the degree of conservation within the sequence were also found.

### Phylogenetic Distances Calculation

**FIGURE 4:**
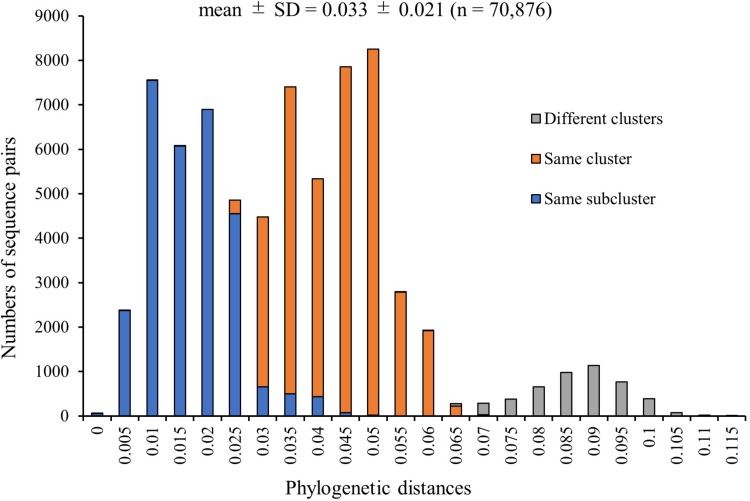
Distribution of phylogenetic distances between the full-length sequences of the *F* gene of all HRV3 strains. The *y*-axis and *x*-axis indicate the number of sequence pairs and phylogenetic distances, respectively. Each colored bar represents the pairs to which the strains belonging: gray, different clusters; orange, same cluster; and blue, same subcluster.

We calculated the phylogenetic distances between the strains based on the nucleotide sequence. The mean ± SD distance of the pairs in the total 377 HRV3 *F* gene strains examined in this study was 0.033 ± 0.021. We plotted the sequence pairs against the distances as a histogram (Figure 4), and it revealed trimodal peaks. Moreover, we colored each pair of the sequences belonging to different clusters (gray), same cluster (orange), and same subcluster (blue). The peaks of these sequence pairs and trimodal peaks of the histogram were consistent, and we also confirmed that the distributions of the three groups were almost completely distinct. Based on the histogram, the cut-off values that classified the sequences belonging to the same subclusters or same clusters and to the same clusters or different clusters were 0.025–0.03 and 0.065–0.07, respectively. These results suggested that the cluster and subcluster classifications were valid in this phylogenetic analysis.

### Selective Pressure Analyses

Positive and negative selection sites of the F protein in all 377 strains were estimated using the Datamonkey web server. Two positive selection sites (aa8 and aa517) were predicted by the MEME method. In contrast, many negative selection sites (388 of 539 amino acid residues, 72.0%) were estimated by the FUBAR method. Moreover, the observed proportion of negative selection sites among the domains of the F protein were not constant (Table 2).

**TABLE 2:** Percentages of negative selection sites for each domain in the F protein.

**Domain of the F protein^∗^**	**Amino acid sequence numbers**	**Negative selection sites (%)**
Signal peptide	1–18	6 sites (33.3)
Fusion peptide	110–135	19 sites (73.1)
Membrane anchor	494–516	12 sites (52.2)
Cytoplasmic tail	517–539	14 sites (60.9)
Domain I (DI)	22–41, 285–368	79 sites (76.0)
Domain II (DII)	375–428	46 sites (85.2)
Domain III (DIII)	42–63, 193–284	83 sites (72.8)
Heptad repeat A (HRA)	129–192	47 sites (73.4)
Heptad repeat B (HRB)	447–484	34 sites (89.5)
Heptad repeat C (HRC)	64–94	21 sites (67.7)
Overall	–	388/539 sites (72.0)

*^∗^The domains of the F protein correspond to the previous study (Yin et al., 2005).*

### Conformational Epitopes and Neutralization-Related Sites Mapped on the Prefusion F Protein Structural Models

**FIGURE 5:**
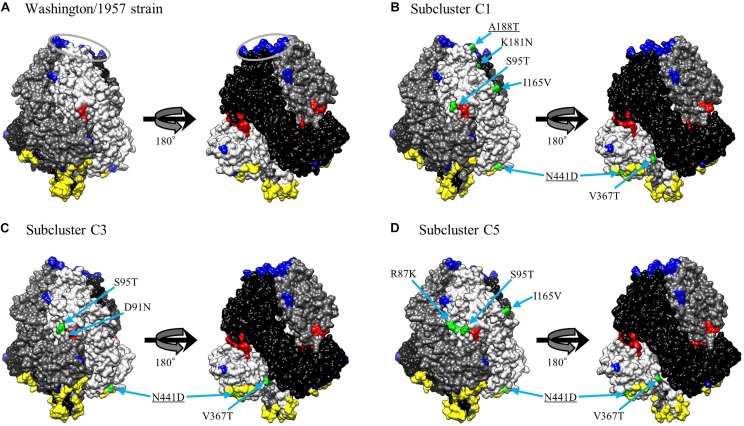
Structural models of the prefusion F protein of Washington/1957 strain **(A)**, subcluster C1 **(B)**, subcluster C3 **(C)**, and subcluster C5 **(D)**. Chains of the trimeric structures are colored in light gray (chain A), dim gray (chain B), and black (chain C). The fusion peptide is shown in red. Conformational epitopes of each strain are indicated in yellow. Previously identified epitopes (MAb binding sites and FAb binding sites) are indicated in blue with the FAb binding sites circled in gray. Amino acid substitution sites of chain A for each variant strain relative to the prototype strain are shown in green. The underlined substitution sites represent the overlap with MAb or FAb binding sites.

We mapped the predicted conformational epitopes and previously identified epitopes with amino acid substitution sites on three-dimensional structure models of the prefusion F protein (Figure 5). The neutralization-related mouse monoclonal antibody (MAb) binding sites and prefusion F-specific antibody (FAb) binding sites previously reported were mapped on the models (Coelingh and Winter, 1990; Stewart-Jones et al., 2018). The conformational epitopes did not correspond to either the MAb or FAb binding sites. Furthermore, none of the substitution sites was found in the neutralization-related sites; these were not therefore inferred to be positively selected. These results suggest that the natural state of the antigenic F protein may not be recognized by antibody-producing human B-cells.

### Amino Acid Residues Observed in the Cleavage Site of the F Protein

Cleavage of the HRV3 F0 protein is critical for membrane fusion, and the amino acid sequences of the cleavage site may be essential for this process. Thus, we analyzed relationships among cleavage sites, F-protein amino acid substitutions, and the selection sites predicted above (Table 3). Residue 108 was the only site of amino acid substitution (Lys108Glu) observed from the prototype strain, with all other amino acids in the cleavage site remaining the same between strains. These cleavage sites other than residue 108 were negatively selected and highly conserved among the collected strains.

**TABLE 3:** The cleavage site sequence of the F protein.

							**cleavage site**	
Residue	104	105	106	107	108	109	↓	110
Negative selection site	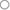	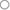	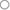	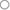		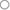	↓	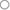
Prototype strain	D	P	R	T	K	R	↓	F
Subcluster C1					E		↓	
Subcluster C3					E		↓	
Subcluster C5					E		↓	

## Discussion

Human respirovirus 3 contains two well-known major antigens, HN and F proteins, which are associated with infectivity, antigenicity, and viral propagation. While some evolutionary studies have focused on the *HN* gene/HN protein, the molecular evolution of the *F* gene/F protein remains unknown at present. Therefore, we performed detailed and comprehensive evolutionary analyses of the HRV3 *F* gene (full-length) in viral strains collected from various areas. This study reveals the following remarkable findings: (i) the time-scaled phylogenetic tree showed that the common ancestor of HRV3 and BRV3 diverged over 300 years ago and subsequently subdivided into three major clusters and four subclusters during the most recent 100 years, (ii) the overall evolutionary rate was approximately 10^–3^ substitutions/site/year, (iii) fluctuations in the effective population size in the gene were observed after the 2000s, (iv) the similarity of the gene was relatively high (>90%) and the mean phylogenetic distance was 0.033 among the strains, (v) most of the amino acid sites were negatively selected, and (vi) the conformational epitopes did not correlate to the neutralizing antibody binding sites of the prefusion F protein. These results suggest that, to protect the protein function, the HRV3 *F* gene remains relatively conserved and the diversity was restricted, although these strains form many branches on the phylogenetic tree (Figure 1). Furthermore, HRV3 reinfection may be responsible for discordances of conformational epitopes and the neutralizing antibody binding sites of the protein. These findings may contribute to a better understanding of HRV3 infection in humans.

Some molecular epidemiological studies of the HRV3 *F* gene have been reported (Godoy et al., 2016; Košutić-Gulija et al., 2017; Tsutsui et al., 2017). Godoy et al. (2016) created a phylogenetic tree based on the partial sequence of the *F* gene (390 nt) by the neighbor-joining method. Moreover, Košutić-Gulija et al. (2017) and Tsutsui et al. (2017) performed a molecular epidemiologic study by the ML method using the full-length nucleotide sequences of the *F* gene from domestic HRV3 strains (Japanese and Croatian strains, respectively). Assessment of viral gene evolutionary rates may help to predict the emergence of new mutants adept at evading host immunity (Motoya et al., 2017). Thus, we performed time-scaled phylogenetic analysis using full-length nucleotide sequences of hundreds of strains collected from various countries and evaluated the dominant type by cluster classification, calculating the evolutionary rates of strains belonging to each cluster and subcluster.

The phylogenetic tree of the *F* gene shown herein demonstrates that HRV3 diverged from a common ancestor with BRV3 about 300 years ago (Figure 1). To our knowledge, it additionally shows for the first time that HRV3 formed three different clusters in the last 100 years, and cluster C, the most prevalent type, formed four subclusters (Figure 1). We concluded that the HRV3 *F* gene sequences belonging to subcluster C4 were lacking in our phylogenetic tree because these strains were unregistered in the GenBank. Our findings for the predicted evolutionary rate of the *F* gene of HRV3 (9.40 × 10^–4^ substitutions/site/year) were identical to those of other RNA viruses, which were predicted to have a nucleotide substitution rate of approximately 10^–3^–10^–4^ (Karron and Collins, 2013). Furthermore, previous reports show similar rates for the HRSV-A and HRSV-B *F* gene (7.59 × 10^–4^ substitutions/site/year and 7.14 × 10^–4^ substitutions/site/year, respectively) (Kimura et al., 2016, 2017). These results suggest that the HRV3 *F* gene evolved in a manner similar to other respiratory viruses such as HRSV. However, it may have a limitation, as we only used the coalescent model as the tree prior model for the phylogenetic analysis using BEAST in this study. A further comparative study of the phylogenetic analyses with different models may be needed.

Next, BSP analyses showed that the overall effective population size of the HRV3 *F* gene fluctuated from the 2000s (Figure 2). Moreover, the population size patterns for subclusters C1, C3, and C5 were roughly similar to that of the total strains. A previous study on human parainfluenza virus infections in hematopoietic stem cell recipients and hematologic malignancy patients revealed an increase of the reports of the infection, and the reason may be the increased awareness and increased availability of fast, economical, reliable diagnostic methods (e.g., real-time polymerase chain reaction; Shah et al., 2016). In relation to this, most of the strains in our dataset were isolated or detected after the 2000s, and this might have affected the diversity of the nucleotide sequences of the gene and the genome population size. There is a peak in the number of samples included in this study around 2010, followed by a rapid decline in the following years (Supplementary Figure S3). Correspondingly, the BSP shows a decrease in effective population size of the F gene around the same time. It is unclear if the decreasing population size is merely a result of an uneven sampling process. For more accurate estimation, an investigation of the underlying incidence and further analyses using a model that can account for fluctuations in the sampling process would be needed (Boskova et al., 2014).

Our similarity analysis for the nucleotide sequences of the *F* gene revealed relatively high similarity (>90%) among the 377 selected strains, consistent with a previous study (Tsutsui et al., 2017). Furthermore, the similarity in some sites including the signal peptide and the junctions of domains were relatively low. These results may affect the present evolutionary rate. Moreover, the HRSV-A and HRSV-B *F* gene also revealed high similarities (>90%) among their strains (Kimura et al., 2016, 2017), suggesting that the *F* genes of HRV3 and HRSV are similarly conserved.

Here, we report a mean phylogenetic distance of 0.033 for the *F* gene from 377 total HRV3 strains (Figure 4), while Tsutsui et al. (2017) calculated a genetic distance of 0.026 in a previous study. This difference between the matrices may be affected by the strain numbers and exclusion of 100% identity strains from our dataset. However, distribution of the distances in our study was similar to those in previous reports of the HRV3 *F* gene (Tsutsui et al., 2017), and also the HRV3 *HN* gene (Mao et al., 2012). This implied that the *F* and *HN* genes may be categorized into the same cluster and subcluster (Godoy et al., 2016; Košutić-Gulija et al., 2017). In addition, the genetic distances of the HRSV-A and HRSV-B *F* gene based on globally collected strains were also smaller than the present study, reported as 0.025 and 0.017, respectively (Kimura et al., 2016, 2017). Taken together, these data suggest that diversities of the *F* gene for these viruses may have similar among and restricted within each species. This may be controlled by individual viral polymerases to protect the functions and structures of the F protein, but further study is needed to explore this possibility. Further, the distributions and cut-off values revealed in this study may help to classify a new isolate in the future.

The selective pressure analyses of the HRV3 F protein by the MEME method estimated two positive selection sites (i.e., aa8 and aa517). The former site belongs to the signal peptide and the latter is within the cytoplasmic tail. The signal peptide gets cleaved during the process of protein maturation via synthesis at the endoplasmic reticulum. It was reported that the peptide contributed to modulate the fusion function of certain paramyxoviruses (von Messling and Cattaneo, 2002). In contrast, the sequence and length of the cytoplasmic tail affected the fusion activity; also, the heterotypic tail from other paramyxoviruses influenced the surface expression levels and activity of the F protein (Zokarkar and Lamb, 2012). However, neither the signal peptide nor the cytoplasmic tail is a component of the ectodomain. Therefore, these sites cannot be the recognition targets of human B-cell, and it is possible that the substitutions in these sites may not be associated with escape mutants (Domingo, 2006).

Negative selection against an amino acid site stabilizes protein functions from deleterious mutations (Domingo, 2006). The fusion peptide of the F protein is an important region to mediate fusion between the virus envelope and the host cell membrane, and this region is highly conserved among the F protein of paramyxoviruses (Donald et al., 2011). Particularly, the glycine residues of the peptide (G112, G116, and G121 in the HRV3 F protein) are considered to play a pivotal role in controlling the activation of the native, metastable form of the F protein (Russell et al., 2004), but these sites were not predicted to be negative selection sites in this study. We also show herein that heptad repeat B (HRB) contains the highest degree of negative selection sites (89.5%). HRB is associated with an “open stalk” formation after the cleavage, and this process causes a refolding of heptad repeat A (HRA) and helps the insertion of the fusion peptide to the target cell membrane (Yin et al., 2006). A previous study reports that some of the amino acid substitutions in this region of the paramyxovirus F protein affected its activation and postfusion six helical bundle (6HB) formation, suggesting that HRB contributes to the conformational switch of the F protein (Russell et al., 2003). In domain II (DII), of which 85.2% is reported herein to be negatively selected, some domain substitutions result in a delay in F-protein activation (Palmer et al., 2012). Additionally, DII has a well conserved region (conserved block of F1, CBF1, consisting of aa381–413 of the HRV3 F protein) across the paramyxovirus family, and this region is considered to be an important site of proper refolding and oligomerization for paramyxovirus F proteins (Gardner et al., 2007). The large number of negative selection sites in regions which mediate the conformational change of the F protein suggests that these domains, rather than other regions predicted *in silico*, have essential roles in the fusion process. This may also be related to the difference in similarities among the regions in the nucleotide sequence of the *F* gene.

Our homology modeling of the F protein prefusion state revealed that existing MAb binding sites, determined by utilizing HRV3 variants (Coelingh and Winter, 1990), and existing FAb binding sites, where the PIA174 antibody binds (Stewart-Jones et al., 2018), do not match predicted conformational epitopes (Figure 5). Moreover, some of the amino acid substitution sites overlap with previously identified epitopes, but those sites are not positively selected. The discordance of the neutralization sites and conformational B-cell epitopes might contribute to the difficulty for the human immune system to produce effective neutralizing antibodies against the HRV3 F protein. As suggested by others for the HRSV-B F protein (Kimura et al., 2017), this discordance might contribute to the mechanism of HRV3 natural reinfection. Verifying this hypothesis will require further *in vitro* and *in vivo* studies.

The proteolytic enzyme that acts on the cleavage site of the F protein cleaves the R-F (Arg-Phe) bond of the F0 protein and generates the F1 and F2 subunits (Chang and Dutch, 2012; Karron and Collins, 2013). This process is essential for viral infection, and we therefore investigated the relationships between the specific amino acid substitutions in the cleavage site of the major prevalent cluster C strains and their infectivity or antigenicity. Recognition by this protease requires the general sequences of aa106-R-X-X-R in the HRV3 F protein just upstream of the cleavage site (Klenk and Garten, 1994). Hence, we evaluated amino acid residues in the activating proteolytic site of the F protein (Table 3). K108E was the only amino acid substitution that was not predicted to be a negative selection site around the region. This substitution was also observed in the strains of cluster B; however, a previous study indicated that the E108 mutation does not affect virus replication or cleavage ability (Coelingh and Winter, 1990). Moreover, the sequences including R106 and R109 are highly conserved in the strains of our present study, excepting the prototype strain, and most sites are negatively selected. These sites are assumed to have no relationship to virulence (Coelingh and Winter, 1990), and no specific substitutions were found in this study. Thus, these results imply that the cleavage site and the comprising amino acid residues do not contribute to the prosperity of the cluster. To our knowledge, this is the first observation to evaluate the preservation of this cleavage site in globally collected HRV3 strains. In addition, these conserved sequences present ideal target motifs for potential vaccines or protease inhibitors to act universally against HRV3 strains.

## Conclusion

In conclusion, the HRV3 *F* gene has phylogenetically evolved into many clusters and subclusters. The nucleotide sequence is relatively conserved and restricted in this diversity. Furthermore, incompatibility between human B-cell epitopes and the neutralization-related sites may be associated with reinfection of HRV3. These findings could contribute to a better understanding of HRV3 virology.

## Data Availability Statement

All datasets generated for this study are included in the article/Supplementary Material.

## Author Contributions

JA, HK, AR, and HT designed the study. JA, HK, TS, DK, YM, and KN analyzed the data. JA, HK, HI, AR, and HT wrote and supervised the study. All authors read and approved the final version of the manuscript.

## Conflict of Interest

The authors declare that the research was conducted in the absence of any commercial or financial relationships that could be construed as a potential conflict of interest.

## Footnotes

1 https://www.ncbi.nlm.nih.gov/genbank/

2 http://tree.bio.ed.ac.uk/software/tracer/

3 http://tree.bio.ed.ac.uk/software/figtree/

4 http://www.datamonkey.org/

5 https://blast.ncbi.nlm.nih.gov/Blast.cgi
