# Supplementary material for: Molecular Evolution of the Fusion Protein (F) Gene in Human Respirovirus 3
Source: Front Microbiol. 2020 Jan 15;10:3054. doi: 10.3389/fmicb.2019.03054 (PMC6974460; doi:10.3389/fmicb.2019.03054)
Supplement: Supplementary file 2 [file Table_1.docx]

Supplementary Material

Molecular Evolution of the Fusion Protein (*F*) Gene in Human Respirovirus 3

Jumpei Aso, Hirokazu Kimura*, Haruyuki Ishii, Takeshi Saraya, Daisuke Kurai, Yuki Matsushima, Koo Nagasawa, Akihide Ryo, and Hajime Takizawa

*** Correspondence:** Dr. Hirokazu Kimura: h-kimura@paz.ac.jp

# Supplementary Files

**Supplementary File S1.** FASTA file of all nucleotide sequences used in this study. GenBank accession numbers are defined in front of each taxon label.

**Supplementary File S2.** XML file of all HRV3 + BRV3 strains with model parameters according to Supplementary Table S3.

**Supplementary File S3.** XML file of all HRV3 strains with model parameters according to Supplementary Table S3.

**Supplementary File S4.** XML file of strains belonging to cluster C with model parameters according to Supplementary Table S3.

**Supplementary File S5.** XML file of strains belonging to subcluster C1 with model parameters according to Supplementary Table S3.

**Supplementary File S6.** XML file of strains belonging to subcluster C3 with model parameters according to Supplementary Table S3.

**Supplementary File S7.** XML file of strains belonging to subcluster C5 with model parameters according to Supplementary Table S3.

# Supplementary Figures


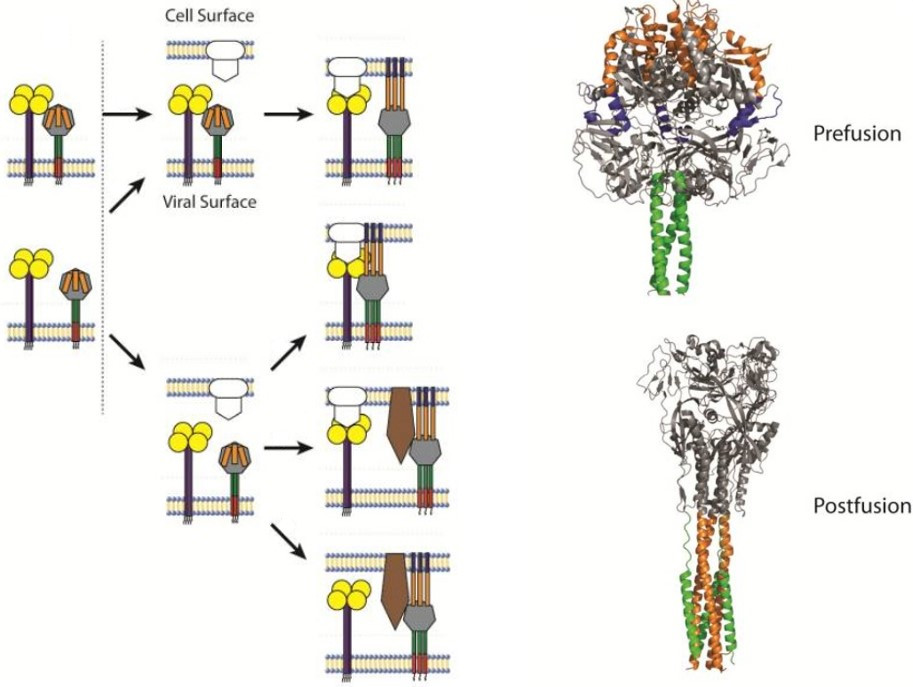


**Supplementary Figure S1.** Illustration of the fusion processes between HRV3 protein and host cellular membranes are shown in the left panel, whereas the 3D structures of the prefusion and postfusion states are shown in the right panel. The detailed processes are described in the text. This figure was adapted and modified from a past work (Chang and Dutch, 2012).


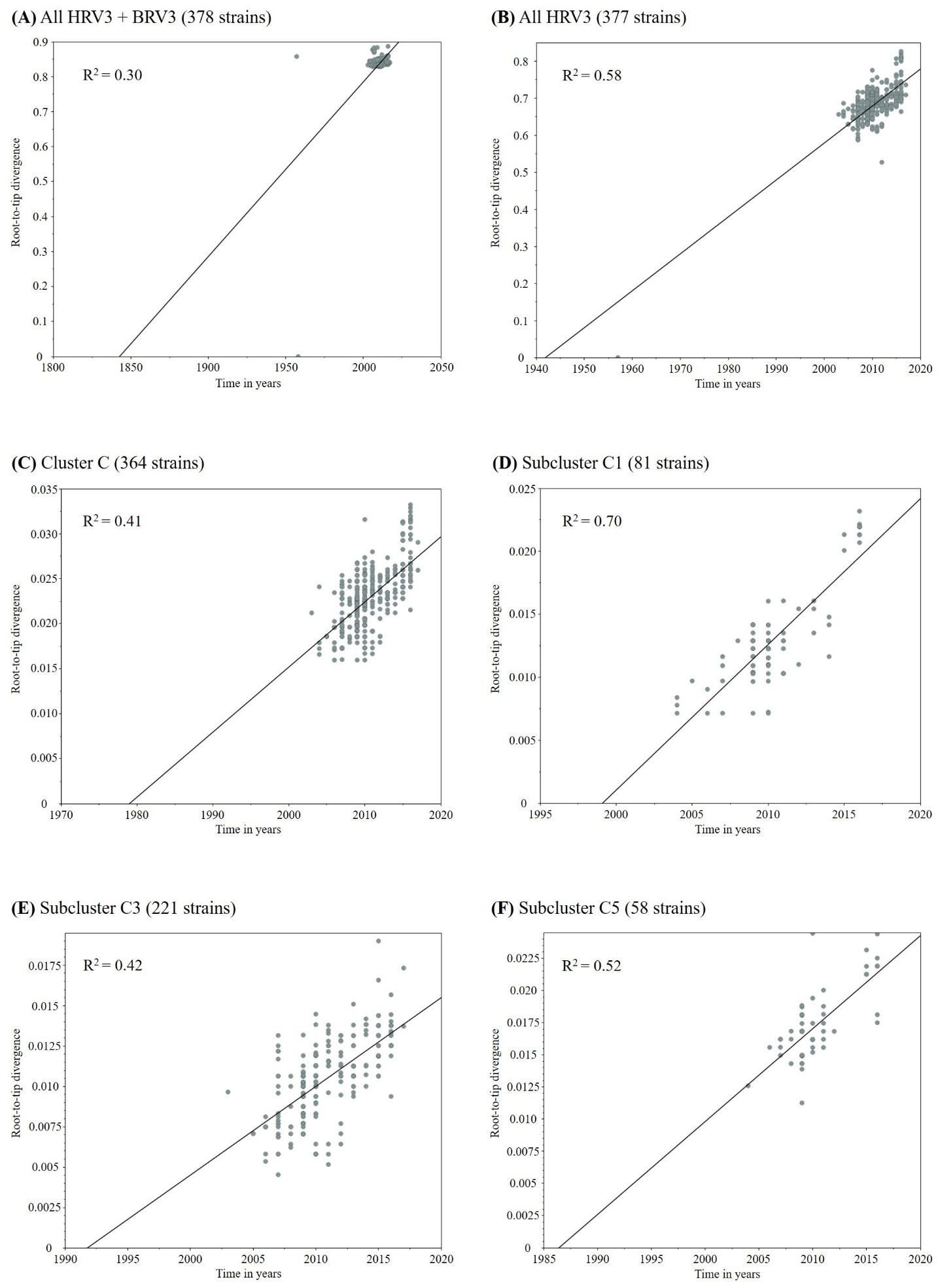


**Supplementary Figure S2.** Root-to-tip regression over time of **(A)** all HRV3 + BRV3 strains, **(B)** all HRV3 strains, **(C)** cluster C, **(D)** subcluster C1, **(E)** subcluster C3, and **(F)** subcluster C5 demonstrated by TempEst version 1.5.3 (Rambaut et al., 2016). R^2^ values show the association between root-to-tip genetic distances and sampling years.


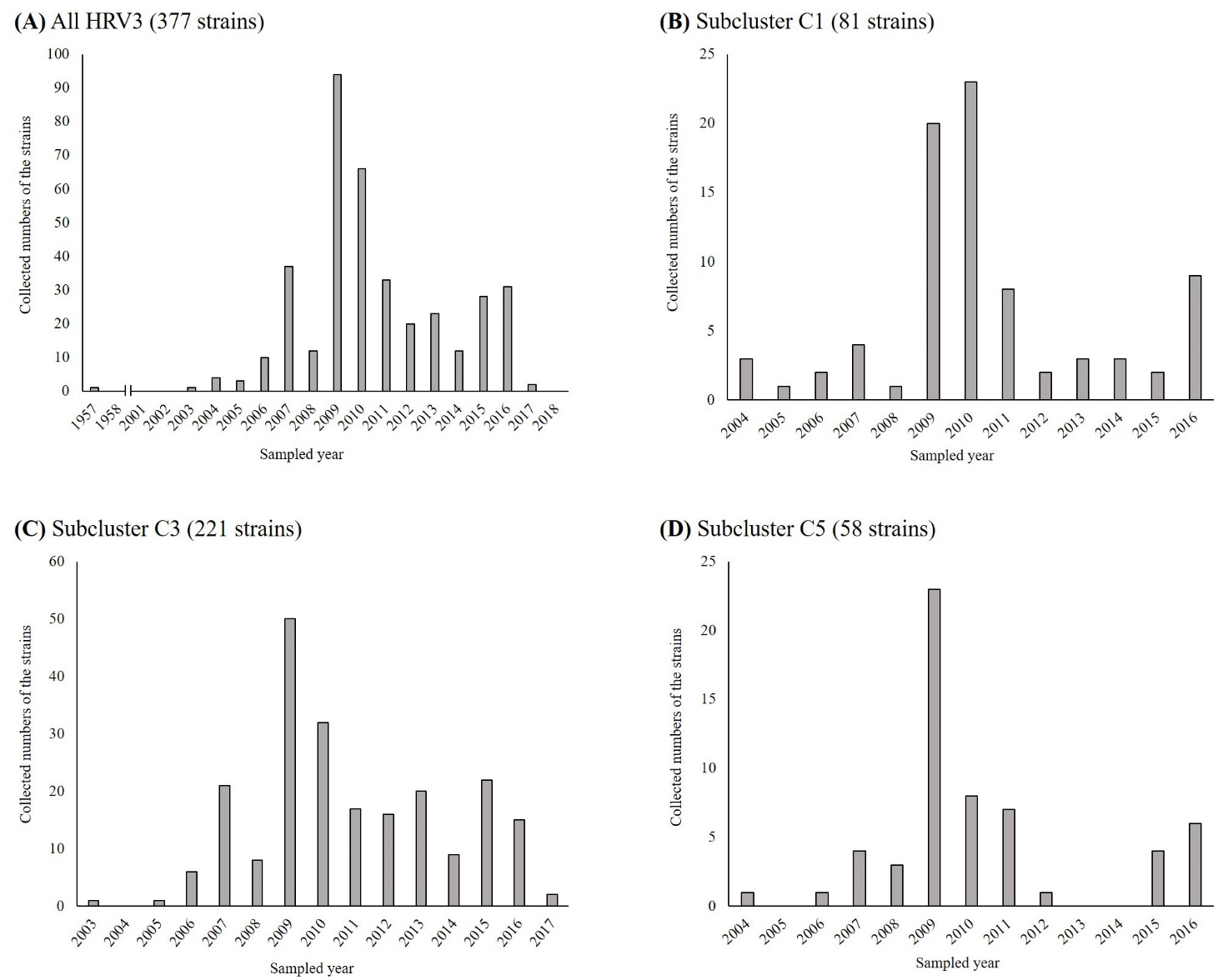


**Supplementary Figure S3.** Histograms of the strain numbers collected for this study and each sampling year for all 377 strains **(A)**, subcluster C1 **(B)**, subcluster C3 **(C)**, and subcluster C5 **(D)**. X-axis indicates the sampling year, whereas y-axis shows the numbers of collected strains.
